# Development of a machine learning model for prognostic prediction of severe sudden sensorineural hearing loss with hyperbaric oxygen therapy

**DOI:** 10.3389/fneur.2025.1701856

**Published:** 2026-01-12

**Authors:** Hyung-Bon Koo, SiHyeong Noh, DongWook Lim, Jung-Hun Kwon, Chang Won Jeong

**Affiliations:** 1 Department of Otorhinolaryngology-Head and Neck Surgery, Wonkwang University College of Medicine, Iksan, Republic of Korea; 2 STSC Center, Wonkwang University, Iksan, Republic of Korea; 3 Smart Team, Wonkwang University Hospital, Iksan, Republic of Korea

**Keywords:** hyperbaric oxygen therapy, machine learning, predictive modelling, prognosis, sudden sensorineural hearing loss, treatment outcome

## Abstract

**Background:**

Severe sudden sensorineural hearing loss (SSNHL) has heterogeneous causes and variable outcomes, making individualized prognosis difficult. We aimed to develop and evaluate a machine-learning (ML) model to predict recovery in severe SSNHL while treating hyperbaric oxygen therapy (HBOT) as an exposure feature rather than inferring causal treatment effects.

**Methods:**

In a single-center retrospective cohort, we analyzed clinical and audiometric data from 231 in patients with severe SSNHL treated between January 2015 and January 2024. Recovery was defined by Siegel’s criteria; eligibility required ≥70 dB loss and treatment initiation within 1 month of onset. Candidate predictors included demographics, comorbidities, baseline thresholds, time to treatment, and HBOT variables (e.g., session count). We trained a custom multilayer perceptron with 12 input features and compared it with conventional algorithms. Performance was assessed using accuracy, F1 score, precision, recall, and area under the ROC curve (AUC).

**Results:**

Among 231 patients, the custom model achieved 89.36% test accuracy and an AUC of 0.8716, outperforming several conventional methods. Key predictors included age, diabetes, dizziness, and HBOT exposure. Notably, “≥10 HBOT sessions” showed high importance in logistic regression and SVM models, suggesting prognostic relevance of sufficient cumulative HBOT exposure.

**Conclusion:**

Including HBOT information as a feature improved prediction of recovery in severe SSNHL; however, these findings do not establish the therapeutic efficacy of HBOT. The model may support clinician–patient decision-making by providing individualized recovery probabilities. Limitations include the retrospective single-center design, modest sample size, and class imbalance, underscoring the need for external validation and better adjustment for confounding.

## 1 Introduction

Sudden sensorineural hearing loss (SSNHL) is an acute disease with a sudden onset of <3 days, diagnosed in patients with a hearing impairment of ≥30 dB in at least three connected frequencies in pure-tone audiometry (1). The annual prevalence in South Korea is approximately ≥10 cases per 100,000 population; most cases are unilateral, but approximately 5–10% are bilateral (2–4). Various pathogenic factors have been suggested for SSNHL, including viral infection, autoimmune conditions, circulatory disorders, and retrocochlear lesions such as acoustic neuroma or stroke; hence, accurate identification of the etiology is challenging. Considering this heterogeneity, predicting the recovery of the patient is extremely difficult (5–7). Several factors have been found to affect the prognosis of SSNHL, including the severity of the initial hearing loss, age, and accompanying vertigo (8, 9). Even after standard systemic steroid and intratympanic steroid treatment, 30% of patients have permanent hearing loss; thus, research is being conducted on adjunct therapies to improve prognoses (10). Hyperbaric oxygen therapy (HBOT) has been proposed as an adjunctive treatment that improves cochlear oxygenation and microcirculation, potentially reducing ischemic injury (11, 12). The 2019 American Academy of Otolaryngology-Head and Neck Surgery (AAO-HNSF) guidelines for sudden hearing loss recommend considering HBOT alongside systemic steroid therapy within 2 weeks of onset, based on findings from previous studies (13).

With the recent advances in artificial intelligence, various studies have been conducted using machine learning (ML) models to predict the prognosis of various diseases, including SSNHL (14, 15). ML models such as linear regressions, support vector machines (SVMs), multilayer perceptron (MLPs), and deep belief networks have been used with features such as age, baseline hearing level, time from onset to treatment, accompanying symptoms, and underlying diseases to attempt to predict the outcomes of SSNHL. In other studies (9, 16), ML models were developed to predict SSNHL prognoses using not only the patient’s baseline condition at the time of visiting the hospital but also other aspects of treatment, such as the start time and frequency of administration of intratympanic dexamethasone injection (ITDI).

Therefore, this study aimed to develop and evaluate ML-based prognostic models for patients with severe SSNHL by incorporating HBOT-related features alongside conventional prognostic factors. By addressing the gap in the existing literature, this work sought to determine whether the inclusion of treatment-based variables can enhance predictive performance in SSNHL prognosis.

## 2 Methods

### 2.1 Ethics statement

In this study, we defined the hearing recovery group as the patients meeting Siegel’s criteria for “complete recovery” or “partial recovery.” The non-recovery group was defined as those meeting the criteria for “slight improvement” or “no improvement.” Informed consent was obtained from all participants prior to their inclusion in the study. This retrospective study was approved by the Institutional Review Board of Wonkwang University Hospital (IRB No. WKUH 2023–06-004). The requirement for written informed consent was waived because anonymized clinical and audiometric data were used. The study was performed in accordance with the guidelines of the Declaration of Helsinki and the principles of Good Clinical Practice.

### 2.2 Study population and data collection

This retrospective study included patients who visited the Wonkwang University Hospital Otolaryngology Department between January 2015 and January 2024 with a main complaint of hearing loss. Patients were diagnosed with SSNHL and received inpatient treatment. To reduce the risk of misclassification and subjective bias, audiometric evaluations were performed by two experienced audiologists using masking techniques. The diagnostic criterion for SSNHL was sudden hearing loss of at least 30 dB in three or more connected frequencies for at least 3 days. Of these patients, we selected those with severe hearing loss of at least 70 dB, on average, in six-frequency pure-tone audiometry and who had started treatment within 1 month of symptom onset. We defined severe hearing loss as a hearing threshold of ≥70 dB, a commonly recognized criterion for profound hearing impairment. Patients meeting this threshold were considered candidates for more aggressive treatment, including HBOT; therefore, only these patients were included in the study cohort. We excluded patients with bilateral SSNHL, other clear causes of hearing loss (e.g., cerebral infarction or acoustic neuroma), or missing data.

### 2.3 Treatment options and audiometric assessments

**Figure 1:**
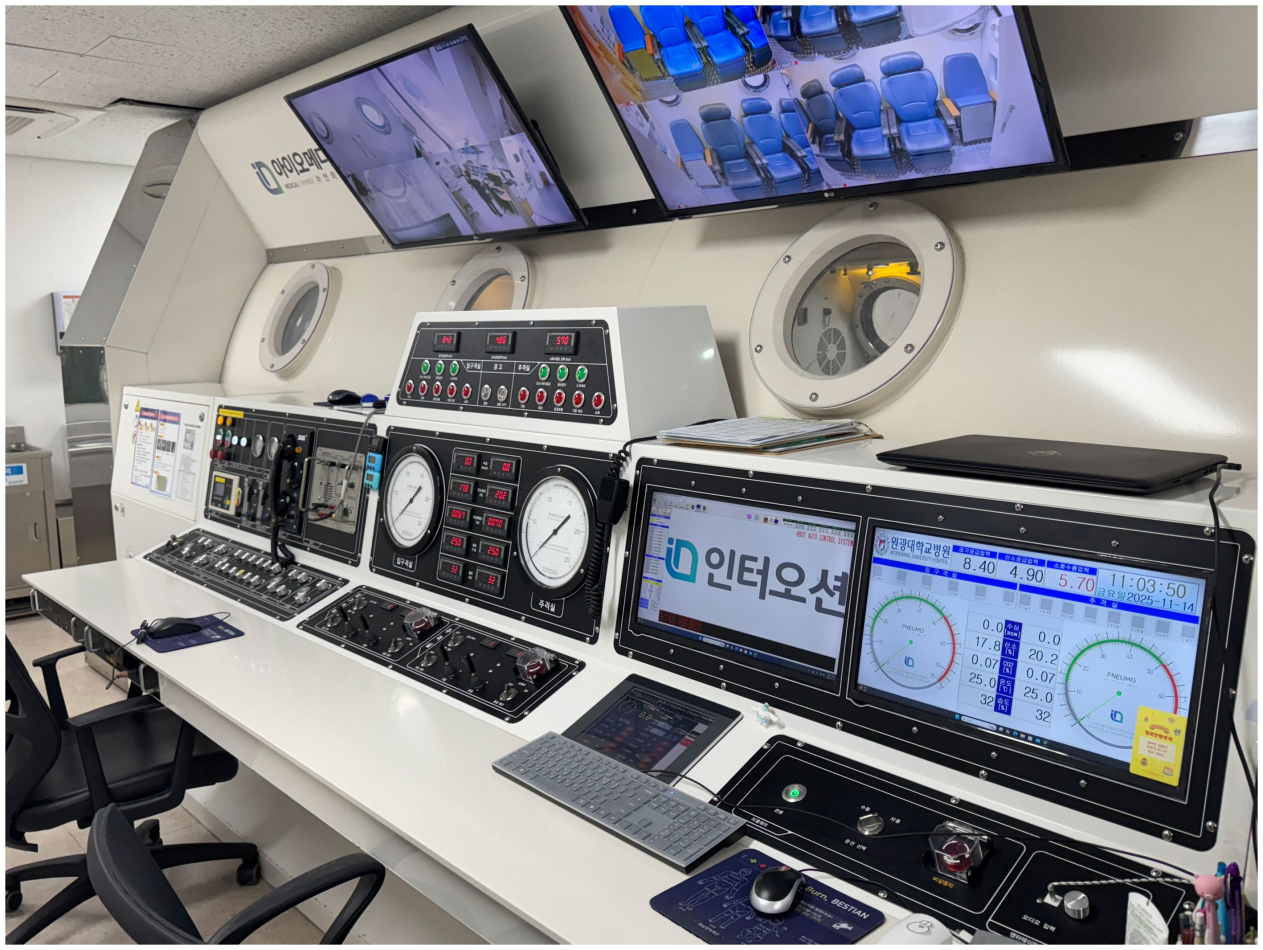
External structures of the hyperbaric oxygen chamber at Wonkwang University Hospital.

**Figure 2:**
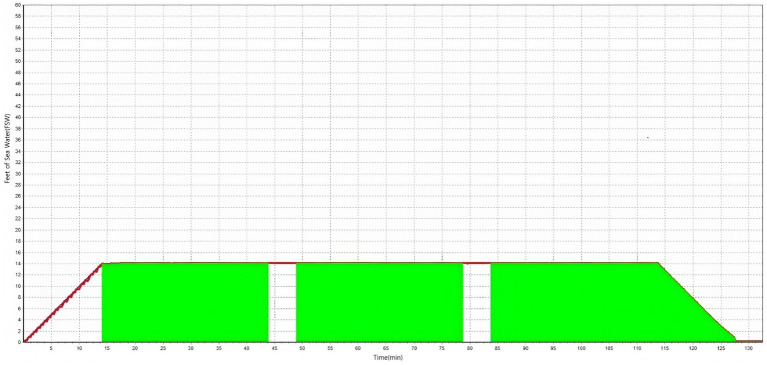
Pressure variation over time (in minutes).

All the included patients received systemic steroids in the form of 1 mg/kg of intravenous prednisolone for 7 days, followed by a 5-day tapering period. For intratympanic steroid injection, 2.5 mg of dexamethasone was administered once per day for 4 days, and another four doses were administered if hearing loss persisted. HBOT was not available for patients who were admitted before March 1, 2020, when the chamber was installed in the hospital. For patients admitted after March 1, 2020, HBOT was administered to patients who chose the treatment after a thorough explanation of its effects and possible side effects (Figure 1). HBOT was performed in the HBOT chamber at 2.8 atm for 114 min daily, starting on the day after admission (Figure 2). After discharge, further treatment was considered for patients who had shown a response after 15–20 sessions and was stopped for patients who showed no response.

During the hospital stay, patients’ physical measurements, disease history, blood tests, and pure-tone audiometry tests were obtained, and pure-tone audiometry was repeated 1 month after treatment. We measured thresholds of hearing at 0.5, 1, 2, and 4 kHz. Siegel’s criteria were classified as follows: complete recovery, final hearing better than 25 dB; partial recovery, improvement >15 dB and final hearing level between 25 and 45 dB; slight recovery, improvement >15 dB and final hearing worse than 45 dB; no improvement, improvement <15 dB or final hearing worse than 75 dB.

### 2.4 Original variables and variable selection

There were initially 40 variables (39 predictor variables and 1 response variable) extracted from demographic data, healthcare records, accompanying symptoms, pure-tone audiometry, and blood tests. There were 13 independent categorical variables: sex, smoking status, drinking status, hypertension, diabetes mellitus, cerebrovascular disease, cardiovascular disease, chronic kidney disease, viral infection, dizziness, tinnitus, use of HBOT, and more or less than 10 HBOT sessions. Apart from sex and more or less than 10 HBOT sessions, the other variables were all binary variables coded as “yes” or “no.”

There were also 28 independent continuous variables: age, height, body weight, body mass index, systolic blood pressure, diastolic blood pressure, glycated hemoglobin (HbA1c), total cholesterol, triglyceride, high-density lipoprotein cholesterol, low-density lipoprotein cholesterol, hemoglobin, erythrocyte sedimentation rate, aPTT, PT results (seconds, percentage, and international normalized ratio), blood urea nitrogen, creatinine, duration from onset to treatment, initial hearing by frequency (0.5, 1, 2, and 4 kHz), six-frequency average, SRT, and frequency of HBOT.

We reduced the feature set to the following 12 variables based on clinical expertise and the importance of the variables across several learning methods: age, HbA1c, viral infection, dizziness, duration from onset to treatment, initial hearing by frequency (0.5, 1, 2, and 4 kHz), six-frequency average, use of HBOT, and more or less than 10 HBOT sessions. The HbA1c data of some patients were not measured; thus, the data were imputed using a multiple imputation method within the normal range (in %) in the data preprocessing process. All other data were suitable for learning, and no other preprocessing was performed.

### 2.5 Statistical analyses and model development

From January 2015 to January 2024, we registered a total of 231 patients with severe SSNHL. Of these, 63 and 168 were in the hearing recovery and non-recovery groups, respectively. We used SPSS version 27.0 (IBM Corp., Armonk, NY, United States) to analyze the two groups statistically. To investigate differences between the recovery and non-recovery groups, we used two-sample *t*-tests for the continuous variables and chi-square tests for the categorical variables. Differences were considered statistically significant if the two-tailed *p* < 0.05 (Table 1).

**Table 1:** Clinical characteristics of patients with sudden sensorineural hearing loss according to hearing recovery.

Variable	Recovery (*n* = 63)	Non-recovery (*n* = 168)	*p*
Age (years)	54.67 ± 17.65	61.46 ± 14.49	0.0032*
Sex	58.06:41.94	58.10:41.90	1.00
Hypertension	38.71:61.29	52.38:47.62	0.098
Diabetes mellitus	24.19:75.81	42.07:57.93	0.022*
Brain disease	9.68:90.32	19.44:80.56	0.127
Heart disease	8.06:91.94	17.48:82.52	0.124
Chronic kidney disease	3.23:96.77	7.64:92.36	0.378
Height (cm)	164.69 ± 9.38	162.48 ± 9.21	0.109
Weight (kg)	67.43 ± 16.57	64.36 ± 11.96	0.123
BMI	24.48 ± 4.32	24.35 ± 3.59	0.619
SBP (mmHg)	123.51 ± 11.57	126.28 ± 15.11	0.185
DBP (mmHg)	74.78 ± 8.64	76.82 ± 10.25	0.162
Alcohol	25.84:74.19	25.87:74.13	1.00
Smoking	17.74:82.26	28.37:71.63	0.152
HbA1c (mmol/L)	7.05 ± 1.77	7.00 ± 1.75	0.924
Total cholesterol (mg/dL)	184.68 ± 48.25	186.79 ± 47.81	0.767
Triglyceride (mg/dL)	84.44 ± 71.85	84.89 ± 45.69	0.967
HDL (mg/dL)	59.06 ± 18.79	61.83 ± 19.52	0.312
LDL (mg/dL)	112.00 ± 41.85	112.20 ± 40.56	0.973
Hb (g/dL)	14.16 ± 1.87	13.69 ± 1.70	0.071
ESR (mm/h)	10.69 ± 16.57	9.11 ± 8.97	0.515
aPTT (s)	29.62 ± 5.79	28.88 ± 3.56	0.388
PT (s)	11.57 ± 2.36	11.39 ± 0.86	0.58
PT (%)	97.23 ± 23.32	104.46 ± 17.03	0.023*
PT (INR)	1.05 ± 0.22	1.04 ± 0.15	0.628
BUN (mg/dL)	17.33 ± 9.31	19.14 ± 9.31	0.185
Creatinine (mg/dL)	0.87 ± 0.31	1.01 ± 0.85	0.21
Virus	3.23:96.77	2.86:97.14	1.00
Dizziness	14.52:85.48	55.00:45.00	1.86e-07
Tinnitus	67.74:32.26	62.86:37.14	0.611
Date	3.22 ± 2.34	3.02 ± 3.89	0.719
Hearing test frequencies			
0.5 kHz	86.58 ± 13.49	90.24 ± 13.89	0.0148*
1 kHz	87.82 ± 10.93	92.63 ± 10.16	0.0002*
2 kHz	79.87 ± 9.91	91.58 ± 10.75	1.20×10^-12^*
4 kHz	77.50 ± 12.69	90.10 ± 10.43	1.47×10^-12^*
Initial average hearing threshold (dB)	80.87 ± 8.44	92.41 ± 8.98	1.55×10^-11^*
SRT (dB)	88.10 ± 11.48	96.43 ± 8.63	0.00001*
HBOT presence	48.39:51.61	44.91:55.07	0.764
HBOT number of times	2.25 ± 5.01	8.26 ± 11.37	2.70×10^-7^*
HBOT 10 times	80.00:20.00	45.71:54.29	0.0819

Values are presented as mean ± standard deviation or number (%). Variables that showed statistical significance are presented in bold type. Statistical significance: *p < 0.05. BMI, body mass index; SBP, systolic blood pressure; DBP, diastolic blood pressure; HbA1c, glycated hemoglobin; HDL, high-density lipoprotein; LDL, low-density lipoprotein; Hb, hemoglobin; ESR, erythrocyte sedimentation rate; aPTT, activated partial thromboplastin time; PT, prothrombin time; INR, international normalized ratio; BUN, blood urea nitrogen; SRT, speech reception threshold; HBOT, hyperbaric oxygen therapy.

The ML model was a custom feedforward neural network constructed using the Keras Sequential API. The network consists of four layers: the first layer is a dense layer with 36 neurons, accepting an input vector of 12 features, and uses the ReLU activation function to introduce non-linearity. The second layer is a Dense layer with 28 neurons, also employing ReLU activation, which helps in learning complex patterns while maintaining computational efficiency. The third layer comprises nine neurons with ReLU activation, further refining feature representations. The final output layer is a single neuron with a sigmoid activation function, providing a probability score for binary classification (e.g., presence or absence of a condition).

Models with up to eight layers were tested, but yielded lower accuracy. We sought to find the model with the highest performance by stacking four to eight layers and adjusting neurons and features. In addition, learning was conducted to create a high-performance model by modifying each of the parameters in Table 2 and optimizing the gamma value inside the loss function.

**Table 2:** Parameters used to train the model for predicting hearing recovery in patients with sudden hearing loss.

Hyperparameter	Value
Batch size	10
Epoch	800
Optimizer	Nadam
Step	80
Hidden layer	2
Loss function	Binary focal cross-entropy

We assessed training outcomes by measuring five indices (accuracy, F1 score, precision, recall, and AUC) for both the training and the test sets. The results are presented in Table 3.

**Table 3:** Results of custom model training.

Metrics	Training results	Test results
Accuracy	0.9783	0.8936
F1 score	0.9750	0.9510
Precision	0.9674	0.9423
Recall	0.9833	0.9600
Area under the curve	0.9965	0.8716

## 3 Results

### 3.1 Clinical characteristics of patients according to their recovery status

A total of 231 patients with severe SSNHL were enrolled in this study, comprising 131 men and 100 women. Among them, 62 patients achieved recovery, while 167 did not. The data demonstrated a notable imbalance between the recovery and non-recovery groups, with the non-recovery group being significantly predominant. The mean ages (±standard deviation [SD]) of the recovery and non-recovery groups were 54.67 ± 17.65 and 61.46 ± 14.49 years, respectively. Table 1 presents the clinical characteristics of the patients depending on their recovery outcomes. Continuous variables are present as mean ± SD, and categorical variables are presented as percentages. Age, diabetes mellitus, prothrombin time (PT; percentage), dizziness, all frequencies in hearing tests, initial average hearing threshold, speech reception threshold (SRT), and HBOT frequency showed statistically significant differences between the recovery and non-recovery groups. Missing data were replaced by the average of normal values.

### 3.2 Model performance results

Table 4 presents data on the comparison of the training and test accuracies of the custom model developed in this study with those of several conventional ML algorithms. The custom model achieved a high training accuracy of 97.83% and an overall good generalization performance, with a test accuracy of 89.36%. In contrast, the decision tree exhibited perfect training accuracy of 100%, but its test accuracy reduced to 78.7%, indicating overfitting. Other algorithms, such as Random Forest, Logistic Regression, Gaussian Naive Bayes, and Support Vector Machine, also achieved high training accuracies but exhibited relatively lower test performances. These findings suggest that the custom model provides more reliable predictive performance in clinical settings compared with traditional ML methods.

**Table 4:** Comparison of the model used in training with other machine learning models.

Training algorithm	Training model accuracy	Test accuracy
Custom model	0.9783	0.8936
Decision tree	1.000	0.787
Random forest	0.864	0.766
Logistic regression	0.864	0.787
Gaussian Naive Bayes	0.788	0.787
Support vector machine	0.810	0.745

The custom model demonstrated strong performance across multiple metrics. During training, it achieved an accuracy of 97.83% and an F1 score of 0.9750, indicating robust classification capabilities. With a precision of 96.74% and a recall of 98.33%, the model efficiently identified positive instances. The AUC was remarkably high at 0.9965, suggesting excellent overall performance. On the test set, performance slightly decreased, with an accuracy of 89.36% and an F1 score of 0.9510. The precision and recall were 94.23 and 96.00% respectively, reflecting reliable yet slightly less stable generalization. The results are shown in Table 3.

**Figure 3:**
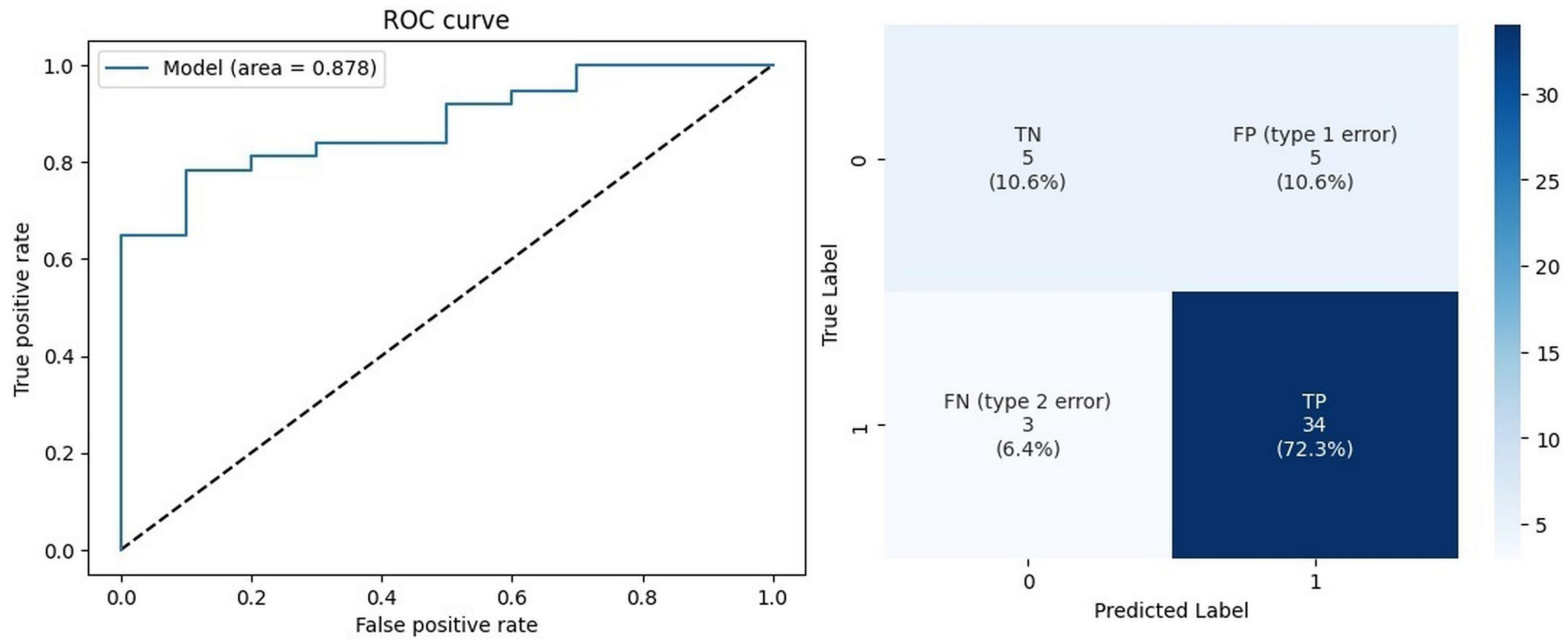
Prediction data outcomes.

Although the decision tree demonstrated marginally better performance on the training set, the custom model achieved superior results on the test set, with approximately 10% higher accuracy. Figure 3 illustrates the receiver operating characteristic (ROC) curve, which reveals the model’s high discriminative ability with an AUC of 0.878, alongside the confusion matrix for the prediction set (data not used during training or testing). Despite the limited number of patients in category 0 (*n* = 63), these results were achieved through extensive parameter tuning involving hundreds of modifications. The ROC curve visually depicts the tradeoff between sensitivity and specificity, while the confusion matrix provides a clear overview of the model’s ability to accurately classify positive and negative cases, highlighting its robustness and potential for clinical application.

## 4 Discussion

This study evaluated the performance of various ML models in predicting hearing recovery among patients with severe SSNHL and poor prognosis, incorporating HBOT administration as a prognostic factor alongside previously established variables. Significant differences between recovery and non-recovery groups were observed across the following variables: age, diabetes, PT (percentage), dizziness, thresholds across all frequencies, initial hearing level, SRT, and HBOT frequency. The custom model achieved a training accuracy of 97.83% and a test accuracy of 89.36%. Although the decision tree demonstrated slightly better performance during training, the custom model outperformed it on the test set, with approximately 10% higher accuracy. Importantly, this study demonstrates the predictive value of incorporating HBOT-related data into ML models, rather than demonstrating the direct therapeutic efficacy of HBOT itself. The clinical insight offered by this model extends beyond traditional prognostic methods by providing personalized recovery probabilities. In clinical otology, such a model could serve as a decision-support tool, assisting clinicians in managing patient expectations, counseling them on the potential benefits and required duration of HBOT, and tailoring treatment strategies based on an individualized prognosis. For instance, a patient with a predicted low recovery rate might be counseled earlier about alternative rehabilitative options like hearing aids or cochlear implants.

SSNHL refers to sudden hearing loss of at least 30 dB in three connected frequencies within 3 days. Although various pathogenic factors have been suggested as causes of SSNHL, including viral infection, ischemic injury due to circulatory disorder, trauma, membranous labyrinth injury, autoimmune disease, stress response, and ototoxic drugs, there is no clear cause in approximately 90% of cases (3, 17, 18). Several factors have been shown to affect the prognosis of SSNHL, including severity of hearing loss, vertigo, age, and time until the start of treatment (6, 19). The AAO-HNSF guidelines recommend using systematic steroids for initial treatment within 2 weeks of onset, but 30% of patients still have permanent hearing loss even after appropriate treatment; thus, research is underway to explore additional treatment methods to improve the prognosis (13, 20). Adjunct therapies such as antivirals, vasodilators, anticoagulants, antibiotics, vitamins, and stellate ganglion block have been studied, but none showed clear therapeutic effects. In contrast, HBOT, developed on the basis that it could reduce ischemic injury by ameliorating inner ear circulatory problems, has been shown to be more effective than other adjunct therapies (21).

An HBOT chamber was installed in our hospital in February 2020, and treatment was offered to patients with severe hearing loss ≥70 dB who requested treatment after a thorough explanation of the effects and side effects. HBOT was first introduced by Boerema et al. (22) in 1960 and has since been used to treat diseases such as carbon monoxide poisoning, anaerobic bacterial infection, decompression sickness, acute central retinal arterial occlusion, burns, and diabetic foot ulcers (23, 24). Ischemic damage of the cochlea due to vascular compromise is thought to cause sudden hearing loss in some cases, and hypoxia due to ischemic injury is believed to be the final pathophysiological process of the disease (25, 26). Based on this theory, HBOT is used as a means of increasing the partial pressure of oxygen (PPO_2_) in tissues by supplying 100% oxygen for approximately 2 h in a specialized chamber at a pressure of 2–3 atm. The higher PPO_2_ increases hemoglobin saturation, increasing the amount of dissolved oxygen, which enables more oxygen supply to the cochlear tissue, mitigating ischemic injury (13). There have been continual efforts internationally to demonstrate the effects of HBOT in SSNHL treatment. According to a review by the Undersea and Hyperbaric Medical Society in 2012, the mean hearing gain in patients with moderate sudden hearing loss was 19.3 dB, and in patients with severe sudden hearing loss, it was 37.7 dB (27). In a meta-analysis including randomized controlled trials conducted by Joshua et al. in 2022, HBOT showed a significant effect in improving hearing (28).

Recently, various ML models have been used to predict hearing outcomes for patients with SSNHL. Suzuki et al. constructed a model to predict hearing recovery after SSNHL treatment using simple and multiple regression analysis and were able to predict hearing improvement with 70% accuracy (29). In the study of Park et al. (16), SVM, random forest, MLP, AdaBoost, and k-nearest neighbor models were used, and the SVM model predicted the hearing prognosis of patients diagnosed with SSNHL with an accuracy of 75.36% (16). In the study by Uhm et al. (8), a model using a deep neural network showed a high accuracy of 88.81% and an area under the curve (AUC) of 0.9448. Previous references have used ITDI and steroids as standard treatments for patients diagnosed with SSNHL, and several other studies have used ginkgo or lipo-prostaglandin as adjunct therapies (8, 9, 14–16). Our study is important because, to our knowledge, it is the first to include HBOT as a prognostic factor, alongside the standard treatment using ITDI and steroids, to predict the hearing prognosis of patients with SSNHL. In models using logistic regression and SVMs, a variable defined as whether or not the patient received ≥10 HBOT sessions showed high importance for predicting hearing improvement. A meta-analysis in 2018 reported that a total treatment time of at least 1,200 min was required to observe the effect of HBOT (12). Accordingly, we used a threshold value of 10 sessions, which is close to 1,200 min, following the protocol at our hospital.

To clarify the quantitative relationship between HBOT and hearing recovery, additional subgroup and regression analyses were performed. Among patients who underwent HBOT, recovery was achieved in 80.0% of those who received ≥10 sessions compared with 45.7% in those receiving <10 sessions, although the difference did not reach statistical significance (*p* = 0.0819). Further analysis confirmed “≥10 HBOT sessions” as a top predictor in both logistic and SVM models, suggesting that sufficient cumulative exposure to HBOT may have prognostic relevance. However, the inverse association observed with overall HBOT frequency likely reflects treatment extension in slow responders rather than direct therapeutic benefit. Therefore, our findings demonstrate the predictive association between HBOT exposure and recovery, rather than a causal therapeutic effect. Future studies should validate these trends using multivariate or time-dependent analyses that adjust for baseline hearing severity and early response status.

We observed significant differences between the recovery and non-recovery groups across various factors (Table 1). First, the non-recovery group had a higher mean age and a greater prevalence of diabetes than the recovery group. This is similar to previous studies, which suggest that weaker immune mechanisms and endothelial abnormalities associated with aging or diabetes may affect the prognosis of SSNHL (1, 8, 30, 31).

In the blood test results, PT (percentage) was significantly higher in the non-recovery group. The labyrinthine artery, a major vessel supplying the inner ear, lacks collateral circulation; thus, clot formation within the artery can lead to ischemia or microcirculatory dysfunction, resulting in sudden hearing loss (32). Furthermore, the cochlea possesses a highly delicate and terminal microvascular system, rendering it particularly vulnerable to even transient reductions in blood flow (32). Based on these reports, clotting status has been hypothesized to be closely related to sudden hearing loss prognosis. In another study, patients with poor response to treatment for sudden hearing loss were found to have significantly lower activated partial thromboplastin time (aPTT) and PT (seconds), along with significantly higher fibrinogen and D-dimer levels (33).

Regarding the initial otolaryngological symptoms related to SSNHL, more patients in the non-recovery group experienced dizziness, whereas no significant difference in the incidence of tinnitus was observed between the recovery and non-recovery groups. Dizziness may imply an injury to the vestibular organ in the inner ear, suggesting that the damage may have extended beyond the auditory nerve to include the vestibular nerve, thereby worsening prognosis (31, 34). Dizziness may also suggest central nervous system involvement, especially certain brain regions such as the brainstem; thus, warranting differential diagnosis (34).

Regarding the initial hearing thresholds, the non-recovery group showed higher results than the recovery group across all frequencies, for the six-frequency method, and SRT. As reported in previous studies, more severe hearing loss was associated with lower recovery rates, suggesting that extensive inflammation or damage in the inner ear could lead to slower hearing recovery (1, 35). Additionally, for HBOT, we observed a significant difference in the performance of HBOT between the recovery and non-recovery groups. The non-recovery group had a higher total number of HBOT sessions than the recovery group. This may be attributed to early discontinuation of HBOT treatment upon hearing improvement, which could have affected the statistical outcomes. As the degree of recovery after treatment may influence this variable in a reverse causal direction, considering HBOT frequency as a true prognostic predictor is challenging. Moreover, this study did not include formal quantitative control for potential confounders such as baseline hearing severity or early treatment response. Given the retrospective design and dataset imbalance, sensitivity analysis or regression adjustments were not feasible. Therefore, the higher HBOT sessions observed in the non-recovery group likely reflect treatment extension in patients with slower improvement rather than an independent prognostic effect. To address this potential confounder, future studies should incorporate methods such as time-dependent covariate analysis or propensity score matching. Given that HBOT frequency protocols have not been clearly established, these findings could guide future studies.

Recent studies have reported the effectiveness of HBOT in cases of SSNHL resulting from high-intensity noise exposure or blast trauma. In particular, some studies have shown that patients with noise-induced SSNHL experienced significant improvement in pure-tone audiometry following combined treatment with steroids and HBOT (36, 37). Ahmed et al. (36) reported a mean hearing improvement of 7 dB in pure-tone average, with greater gains of 12.4 dB specifically at 4 and 8 kHz following treatment. However, the present study did not include such etiologies, and future research should consider these specific causes to enhance the clinical applicability of the findings. Additionally, applying the same ML model without incorporating HBOT as a variable and comparing it with the current model may further clarify the therapeutic value of HBOT.

Adverse effects were observed in 24 patients, which is a relatively large proportion (25%) of the 96 patients who underwent HBOT. However, no patients experienced severe or permanent complications, and all recovered after appropriate monitoring. Of the 24 patients who experienced adverse effects, 21 (87.5%) showed otological symptoms or findings, such as otalgia, hemotympanum, dizziness, or tympanic membrane deformation. Two patients (8.3%) complained of stuffiness in the chest, but no specific findings were observed when examined. One patient (4.2%) complained of myopia, but recovered normal vision 1 month after the onset of symptoms. The most representative adverse effect of HBOT is pressure injury to the middle ear. This is typically prevented using the Valsalva maneuver to regulate the pressure between the middle and outer ear, but in patients with Eustachian tube dysfunction or reduced performance ability for the Valsalva maneuver, pressure differences can cause injury to the tympanic membrane or middle ear. Other potential adverse effects, which we did not observe in our study, are pressure injury to the paranasal sinuses, which is relatively common, and, more rarely, muscle spasms due to oxygen toxicity (38, 39). Additionally, some patients may complain of claustrophobia in the confined space of the chamber; thus, this should be verified during history-taking.

A limitation of this study is the small sample size, with only 231 patients. We believe this is due to the strict criterion indicating that only patients with severe SSNHL (≥80 dB) can receive insurance coverage for HBOT in South Korea. Considering that the study cohort was derived from a single tertiary center in Korea and included only 231 patients, external validation using a multicenter or international dataset is warranted to ensure generalizability and broader applicability of the model. Additionally, some patients could have experienced hearing improvements after the follow-up period. Most cases of hearing improvement are known to occur within 2 months, but rarely some patients recover hearing even later than 2 months (40, 41). The protocol at our hospital dictates that follow-up is stopped after 2 months; hence, we were unable to verify later changes. Although some patients are still under observation, most were lost to follow-up. In cases of non-recovery, future research should focus on establishing treatment protocols that incorporate hearing aids, bone-anchored hearing aids, or cochlear implants to optimize therapeutic outcomes. In future research, we aim to recruit more patients and provide better control using objective examinations and diverse treatment methods. It is important to note that the retrospective design of this study and the use of historical control comparisons may introduce limitations in the interpretation of the results. To address insufficient and unbalanced datasets, we plan to conduct learning through cross-validation methods such as stratified K-fold. Additionally, the audiometric testing protocol at our institution is restricted to hearing frequencies up to 4 kHz, thereby preventing assessment of higher frequencies, thus representing a limitation of this study.

In conclusion, we demonstrated that the custom ML model effectively predicts hearing recovery in severe SSNHL with poor prognosis, achieving high accuracy in both training and testing. While the decision tree showed marginally better training performance, the custom model achieved superior results on the test set, with about 10% higher accuracy. As protocols for HBOT frequency remain undefined, these findings may inform future research directions.

## Data Availability

The datasets presented in this study can be found in online repositories. The names of the repository/repositories and accession number(s) can be found in the article/Supplementary material.
